# Characteristics of the completed chloroplast genome sequence of *Xanthium spinosum*: comparative analyses, identification of mutational hotspots and phylogenetic implications

**DOI:** 10.1186/s12864-020-07219-0

**Published:** 2020-12-02

**Authors:** Gurusamy Raman, Kyu Tae Park, JooHwan Kim, SeonJoo Park

**Affiliations:** 1 Department of Life Sciences, Yeungnam University, Gyeongsan, Gyeongsangbuk-do Republic of Korea 38541; 2 Department of Life Sciences, Gachon University, Seongnam, Gyeonggi-do Republic of Korea

**Keywords:** Nucleotide diversity, Divergence, Ambrosiinae: genetic markers, phylogenomics

## Abstract

**Background:**

The invasive species *Xanthium spinosum* has been used as a traditional Chinese medicine for many years. Unfortunately, no extensive molecular studies of this plant have been conducted.

**Results:**

Here, the complete chloroplast (cp) genome sequence of *X. spinosum* was assembled and analyzed. The cp genome of *X. spinosum* was 152,422 base pairs (bp) in length, with a quadripartite circular structure. The cp genome contained 115 unique genes, including 80 PCGs, 31 tRNA genes, and 4 rRNA genes. Comparative analyses revealed that *X. spinosum* contains a large number of repeats (999 repeats) and 701 SSRs in its cp genome. Fourteen divergences (Π > 0.03) were found in the intergenic spacer regions. Phylogenetic analyses revealed that *Parthenium* is a sister clade to both *Xanthium* and *Ambrosia* and an early-diverging lineage of subtribe Ambrosiinae, although this finding was supported with a very weak bootstrap value.

**Conclusion:**

The identified hotspot regions could be used as molecular markers for resolving phylogenetic relationships and species identification in the genus *Xanthium*.

## Background

The structure of the majority of the flowering plant chloroplast (cp) genome consists of a pair of inverted repeats (IRs), along with large single-copy (LSC) and small single-copy (SSC) regions, and cp genome size ranges from 107 to 280 kb [1, 2]. With the emergence of next-generation sequencing technology [3], complete cp genome sequences are being extensively used to improve phylogenetic resolution at the interspecific level [4]. In addition, cp genomes have been found to contain polymorphic regions generated through genomic expansion, contraction, inversion, or gene rearrangement, and such sequences have been widely used as an effective tool for plant phylogenomic analyses [5].

The invasive species *Xanthium spinosum* belongs to the family Asteraceae and is within the subtribe Ambrosiinae (Heliantheae), which includes annual and perennial herbaceous plants [6]. It is native to South America and has been introduced to Canada, the United States, Central and South America, parts of Africa, the Middle East, Russia, China, Australia, and the Korean Peninsula [7–10]. The genus *Xanthium* has been widely used for various traditional medicinal treatments in multiple countries [11]. Parts of the *X. spinosum* plant are used for the treatment of cancer and diarrhea [12, 13], intermittent fever related to hydrophobia and rabies [14], and rheumatoid arthritis [15], and have antibacterial [14] and antiviral properties [14, 16–18]. Although several antimicrobial substances and their functions have been studied in *X. spinosum* over the past five decades, no exclusive genetic or genomic studies have been conducted to date.

Universal molecular markers such as the plastid genes *rbcL* and *psbA* and nuclear internal transcribed spacer (ITS) have been widely used for the rapid and precise identification of plant species but have proved unsuccessful for distinguishing very closely related species [19–21]. The genus *Xanthium* is commonly known as cocklebur, and is a close relative of the genus *Ambrosia*. The number of species in the genus *Xanthium* remains under debate, and this genus may include 5 to more than 20 species [22–25]. Phylogenetic analyses of several plastid and nuclear DNA markers have shown conflicting results for *Xanthium* and its relatives [11]. By contrast, Somaratne et al. (2019) used 46 cp protein-coding genes (PCGs) to resolve the phylogenetic positions of *Xanthium* and *Parthenium* and revealed that *Parthenium* is not an early-diverging lineage of the subtribe Ambrosiinae. However, most plant cp genomes contain highly conserved structures that are useful molecular markers for the identification of plant species in genome-wide evolutionary studies; such structures provide significant quantities of genetic information and can resolve taxonomic and phylogenetic relationships [26, 27].

In the present study, we examined both plastome evolution and the phylogenetic relationships within Heliantheae. For this purpose, we first sequenced and characterized the *X. spinosum* cp genome and compared it with the *X*. *sibiricum* cp genome as well as those of closely related species of Heliantheae. In addition, we identified hotspot regions of sequence variation and clarified the evolutionary dynamics among *Xanthium* species.

## Results

### General features of the cp genome and its organization

**Fig. 1:**
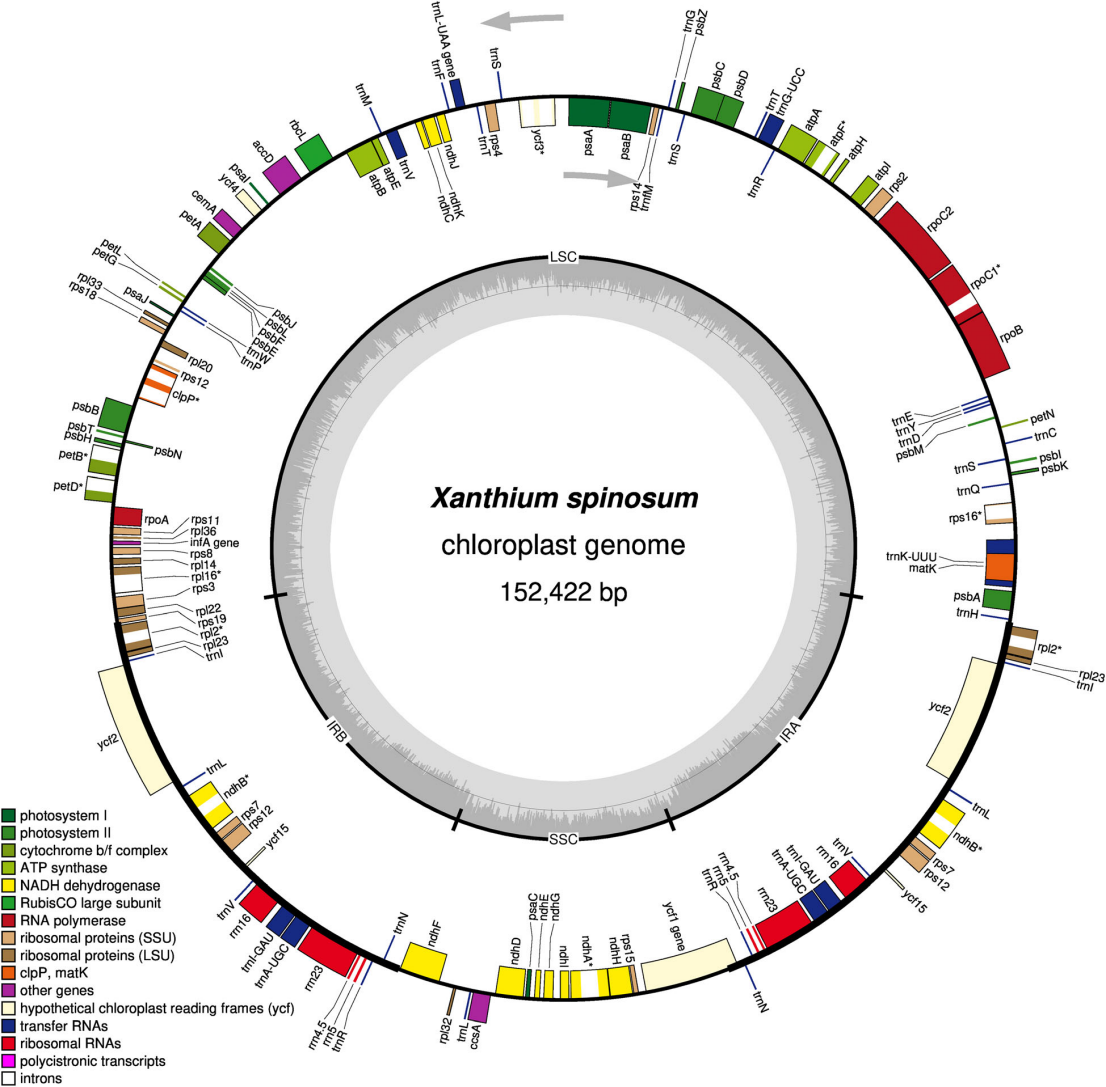
Gene map of *Xanthium spinosum*. Genes lying outside the outer circle are transcribed in a counter-clockwise direction, and genes inside this circle are transcribed in a clockwise direction. The coloured bars indicate known protein-coding genes, transfer RNA genes, and ribosomal RNA genes. The dashed, dark grey area in the inner circle denotes GC content, and the light grey area indicates genome AT content. LSC, large single-copy; SSC, small single-copy; IR, inverted repeat

The complete cp genome of *X. spinosum* was 152,422 bp in length. The cp genome showed a typical quadripartite structure containing two short inverted repeats (IRa and IRb) (25,075 bp) separated by a small single-copy (SSC) region (18,083 bp) and a large single-copy (LSC) region (84,189 bp) (Fig. 1). The cp genome encodes 115 unique genes, including 80 PCGs, 31 transfer RNA (tRNA) genes, and 4 ribosomal RNA (rRNA) genes. Six protein-coding, six tRNA, and four rRNA genes were duplicated in the IR regions. The overall GC content of the cp genome was 37.4%, while those of LSC, SSC, and IR regions were 35.4, 31.2, and 43%, respectively (Table 1).

**Table 1:** Comparative analysis of chloroplast genome of *X*.*spinosum* and *X*. *sibiricum*

Characteristics		***X. spinosum***	***X. sibiricum***
Size (bp)		152,422	151,897
LSC length (bp)		84,189	83,847
SSC length (bp)		18,083	17,900
IR length (bp)		25,075	25,070
Total number of genes		132	132
Protein-coding genes		87	87
tRNA genes		37	37
rRNA genes		8	8
Duplicate genes		17	17
GC content	Total (%)	37.4	37.5
	LSC (%)	35.4	35.5
	SSC (%)	31.2	31.4
	IR (%)	43	43
	CDS (%)	37.9	37.9
	rRNA (%)	55.2	55.2
	tRNA (%)	53	52.9
	All genes (%)	39.5	39.5
Protein-coding genes (%bp)		51.65	51.74
All genes (%bp)		72.5	72.89
Non-coding regions (%)		27.5	27.11

### Comparative analyses of *Xanthium* species

**Fig. 2:**
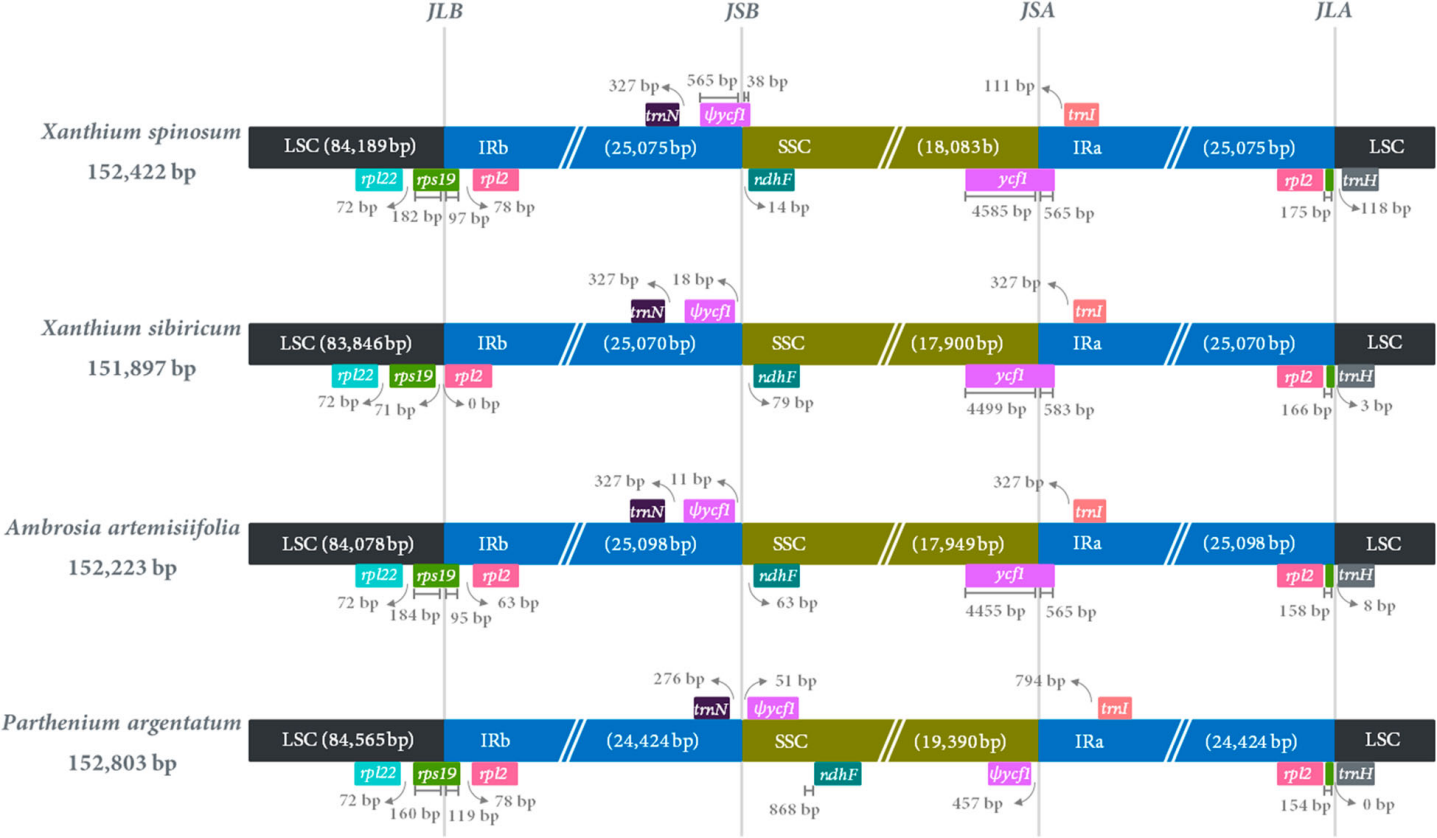
Comparison of the large single-copy (LSC), small single-copy (SSC) and inverted repeat (IR) border regions of four Heliantheae (*Xanthium spinosum*, *X*. *sibiricum*, *Ambrosia artemisiifolia* and *Parthenium argentatum*) chloroplast genomes. Ѱ indicates a pseudogene. The figure is not drawn to scale

The borders of LSC-IRb and SSC-IRa in the cp genome of *X. spinosum* were compared to three other closely related species of Heliantheae, namely, *X*. *sibiricum*, *Ambrosia artemisiifolia*, and *Parthenium argentatum* [28, 29] (Fig. 2). An intact copy of the *rps19* gene was present in the LSC/IRb borders of *X. spinosum*, *A. artemisiifolia*, and *P. argentatum*, as well as a shared 95 bp to 119 bp sequence in the IRb region adjacent to the *rpl2* gene. By contrast, the *X*. *sibiricum rps19* gene was completely shifted to the LSC region, 71 bp away from the IRb region, despite the *rpl2* gene being present at the LSC/IRb border. In addition, 154–175 bp of the fragmented *rps19* gene in all four species was present at the IRa/LSC, LSC/IRa regions or its border. On the other hand, ѱ*ycf1* was present in the IRa/SSC border of *X. spinosum*, whereas it was located in the IRb or silenced in the SSC region of *X*. *sibiricum* and *A. artemisiifolia*, and was situated in the SSC region of the *P. argentatum* cp genome. The entire *ndhF* gene was present in the SSC region of all four cp genomes. Similarly, an intact *ycf1* gene was present in the SSC/IRa region of all of the cp genomes analyzed, except *P. argentatum*, which has a 565 to 583 bp fragment of *ycf1* in the IRa region. However, *P. argentatum* encodes two copies of ѱ*ycf1* in its genome. The *trnH* gene sequences are located in the LSC region 0 to 118 bp from the IRa/LSC border in all cp genomes.

**Fig. 3:**
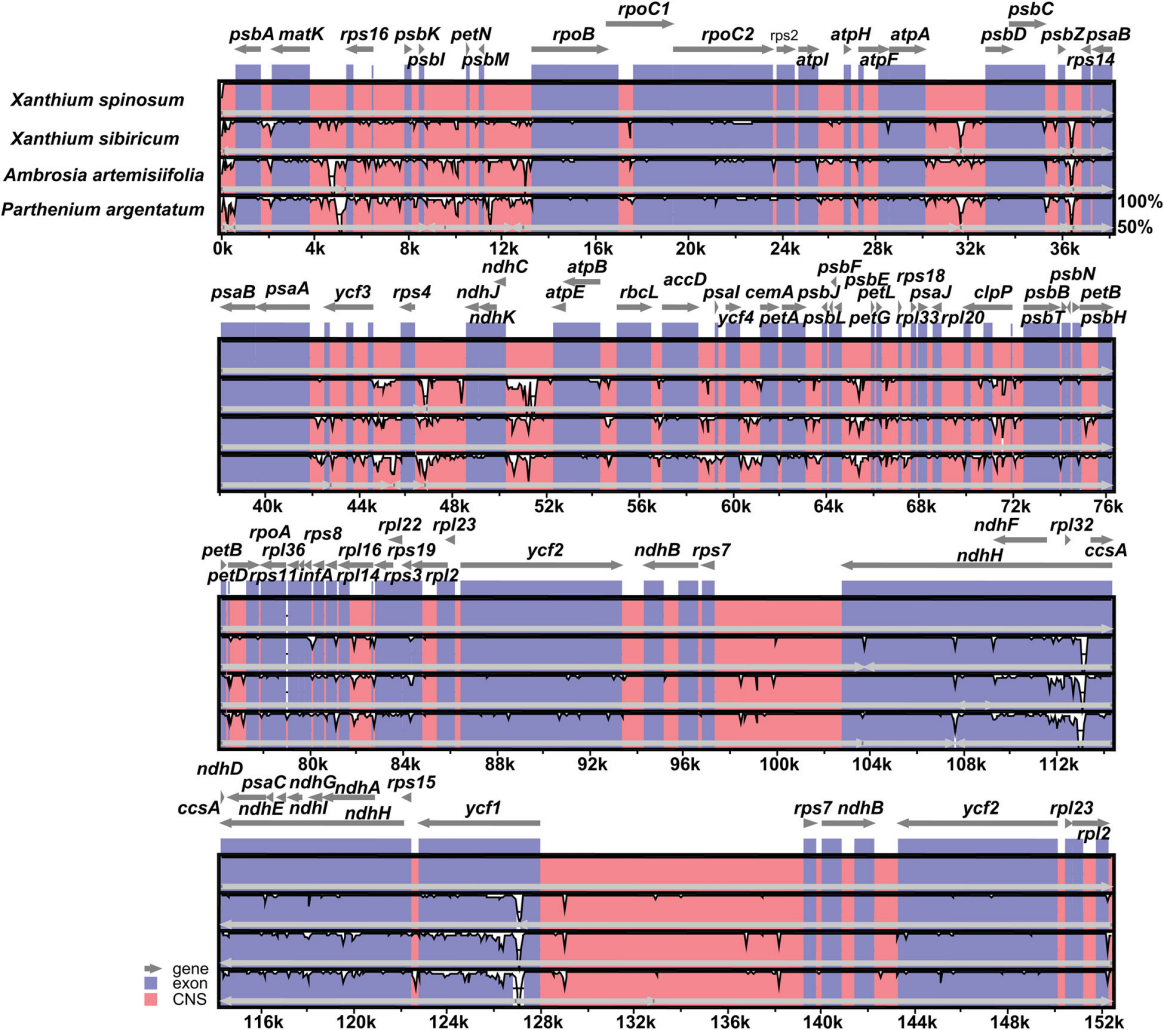
Sequence alignment of four Heliantheae chloroplast genomes performed using the mVISTA program with *Xanthium spinosum* as a reference. The top grey arrow shows genes in order (transcriptional direction) and the position of each gene. A 70% cut-off was used for the plots. The y-axis indicates a percent identity of between 50 and 100%, and the red and blue areas indicate intergenic and genic regions, respectively

The cp genomic sequences of four Heliantheae species were analyzed using mVISTA software to detect variation among the sequences (Fig. 3). The sequence divergence differed markedly among regions. The data revealed that the non-coding region was more divergent than its coding counterparts. Relative to the LSC and SSC regions, IR regions of all cp genomes were less divergent.

### Repeat structure and SSR analyses

**Fig. 4:**
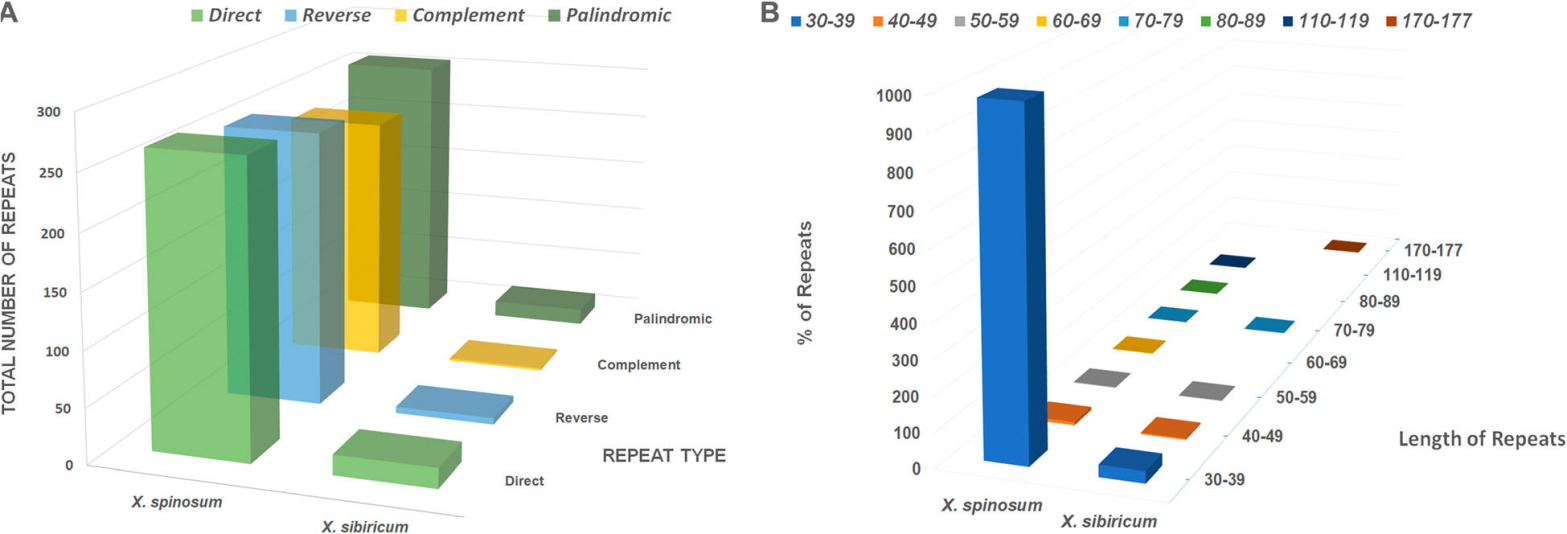
Comparison he distribution of different repeat types in the *Xanthium spinosum* vs. *X*. *sibiricum* cp genomes. **a** The number of different types of repeats. F – forward repeats; R – Reverse repeats; P – palindromic repeats; C – complement repeats. **b** The length and the total number of repeat sequences present in their respective cp genomes

The presence of repeat sequences in the *X. spinosum* and *X*. *sibiricum* cp genomes was analyzed and the species were compared. Repeats in the *X. spinosum* cp genome consist of 264 forward, 256 palindromic, 251 reverse, and 228 complement. By contrast, *X*. *sibiricum* contained 18 forward, 15 palindromic, 6 reverse, and 2 complement repeats (Fig. 4a). In total, *X. spinosum* and *X*. *sibiricum* contain 999 repeats and 41 repeats, respectively. Among the 999 repeats identified in *X. spinosum*, repeats of 30–39 bp in length (983) were predominant in the cp genome; the longest repeat was 115 bp and was a palindrome sequence. Similarly, in *X*. *sibiricum*, 34 repeats were 30–39 bp in length, and the longest was a palindromic sequence of 177 bp (Fig. 4b).

**Fig. 5:**
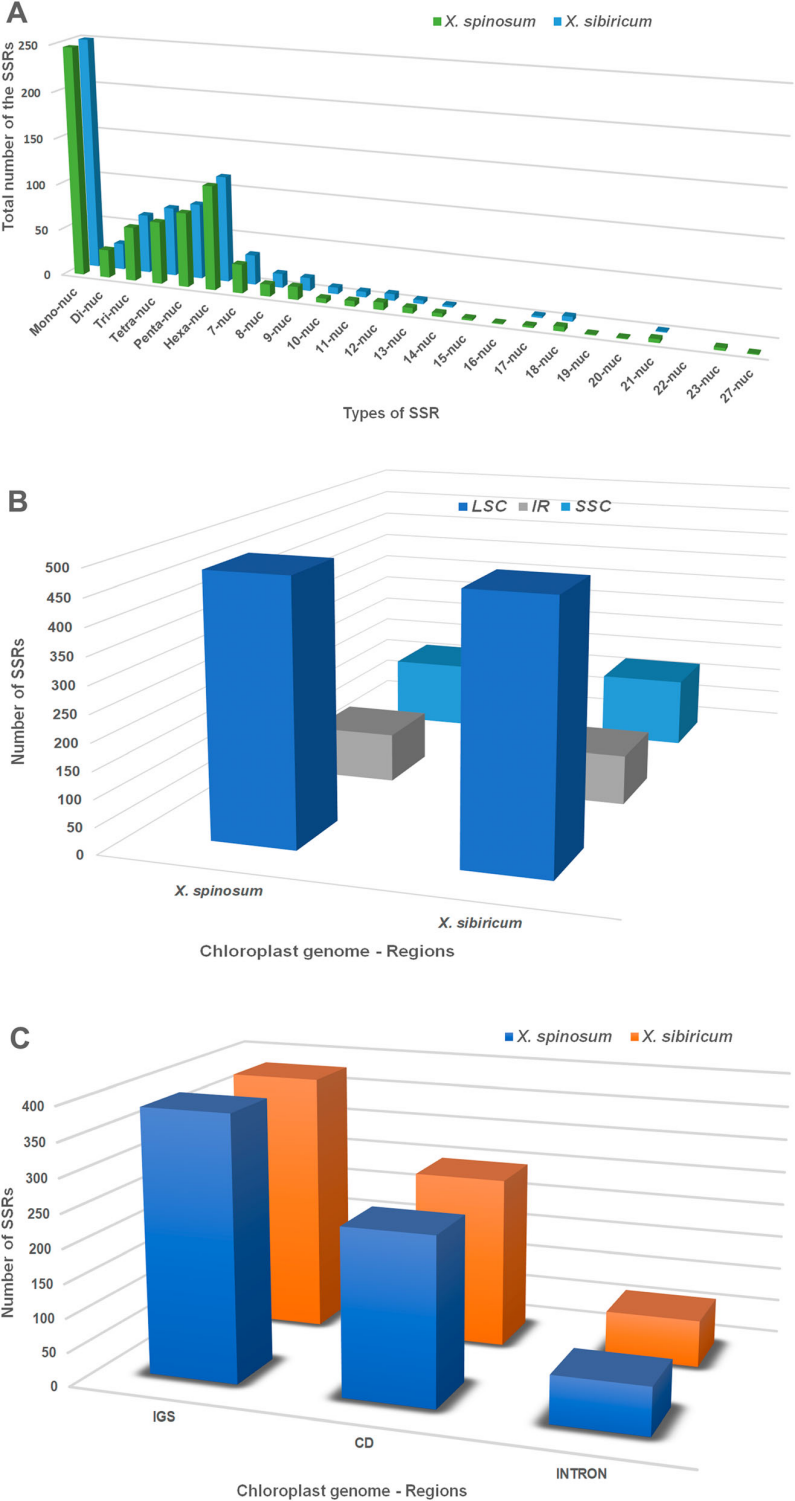
Comparison the presence of simple sequence repeats (SSRs) in the *Xanthium spinosum* vs. *X*. *sibiricum* cp genomes. **a** Distribution of different types of SSRs. **b** Presence of SSRs in the LSC, SSC, and IR regions. **c** Presence of SSRs in intergenic spacers, protein-coding regions, and intron regions

In total, 701 and 705 simple sequence repeats (SSRs) were identified in the *X. spinosum* and *X*. *sibiricum* cp genomes, respectively. The 701 SSRs in the *X. spinosum* cp genome included 247 (35.24%) mono-nucleotide repeats, 30 (4.3%) di-nucleotide repeats, 58 (8.3%) tri-nucleotide repeats, 67 (9.6%) tetra-nucleotide repeats, 80 (11.4%) penta-nucleotide repeats, 112 (15.98%) hexa-nucleotide repeats, 31 (4.42%) 7-nucleotide repeats, and 76 other repeats ranging from 8 nucleotides to 27 nucleotides (10.84%) (Fig. 5a). Similarly, the cp genome of *X*. *sibiricum* contained 250 (35.46%) mono-nucleotide repeats, 28 (3.97%) di-nucleotide repeats, 63 (8.94%) tri-nucleotide repeats, 74 (10.5%) tetra-nucleotide repeats, 81 (11.49%) penta-nucleotide repeats, 114 (16.18%) hexa-nucleotide repeats, 32 (4.54%) 7-nucleotide repeats, and 63 repeats with lengths from 8 nucleotides to 21 nucleotides (8.94%). Furthermore, the distributions of SSRs in the LSC, IR and SSC regions of *X. spinosum* and *X*. *sibiricum* indicated that the corresponding cp genomes contain 483 and 481 SSRs in the LSC, 91 and 93 in the IR, and 127 and 131 in the SSC regions (Fig. 5b). Likewise, SSRs were analyzed in the protein-coding (exon, protein-coding exon), intron and intergenic spacer (IGS) sequences of *X. spinosum* and *X*. *sibiricum*, which indicated that their cp genomes contain 244 and 252 SSRs in CDs, 69 and 69 in introns and 388 and 384 in IGS regions, respectively (Fig. 5c).

### Nucleotide diversity analyses

**Fig. 6:**
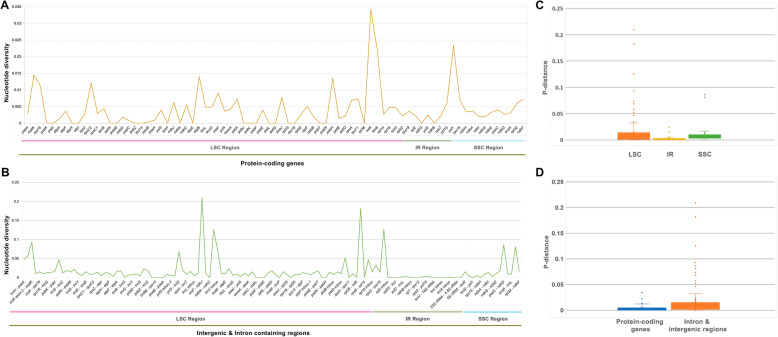
The genetic diversity based on Kimura’s two-parameter model. **a** The P-distance value of protein-coding genes (**b**) the P-distance value of intron and intergenic regions **(c)** Boxplots of P-distance value difference among LSC, IR and SSC regions (**d**) Boxplots of P-distance value differences between protein-coding genes and intron and intergenic regions

The nucleotide diversity of 208 regions was analyzed using DnaSP software, including 79 PCGs and 129 intergenic and intron regions in the cp genomes of *X. spinosum* and *X*. *sibiricum*. The most variable region was *infA* (0.03) among PCGs (Fig. 6a), and high variability was observed for the *trnH*-*psbA* (0.05), *psbA*-*trnK* (0.06), *trnK* exon2-*matK* (0.09), *psbI*-*trnS* (0.05), *ycf3*-*trnS* (0.07), *trnF*-*ndhJ* (0.21), *ndhC*-*trnV* (0.13), *trnV* intron (0.07), *petD*-*rpoA* (0.05), *infA*-*rps8* (0.18), *rpl14*-*rpl16* (0.05), *rpl16*-*rps3* (0.03), *psaC*-*ndhD* (0.09) and *trnL*-*rpl32* (0.08) genes in introns and intergenic regions (Fig. 6b; Table 2).

**Table 2:** Mutational hotspots between *X*. *spinosum* and *X*. *sibiricum*

S. No.	Region	Nucleotide diversity (Pi)	Total number of mutations	Region length (bp)
1	*trnH* - *psbA*	0.04774	19	398
2	*psbA* - *trnK*	0.05714	12	210
3	*trnK* exon 2 - *matK*	0.09286	26	280
4	*psbI* - *trnS*	0.04667	7	150
5	*ycf3* - *trnS*	0.06838	56	819
6	*trnF* - *ndhJ*	0.20940	49	234
7	*ndhC* - *trnV*	0.12551	123	980
8	*trnV* intron	0.07360	29	394
9	*petD* - *rpoA*	0.05181	10	193
10	*infA* - *rps8*	0.18189	22	121
11	*rpl14* - *rpl16*	0.04673	5	107
12	*rpl16* - *rps3*	0.03226	5	155
13	*psaC* - *ndhD*	0.08621	10	116
14	*trnL* - *rpl32*	0.08088	44	544

### Synonymous (K_S_) and nonsynonymous (K_A_) substitution rate analyses

**Fig. 7:**
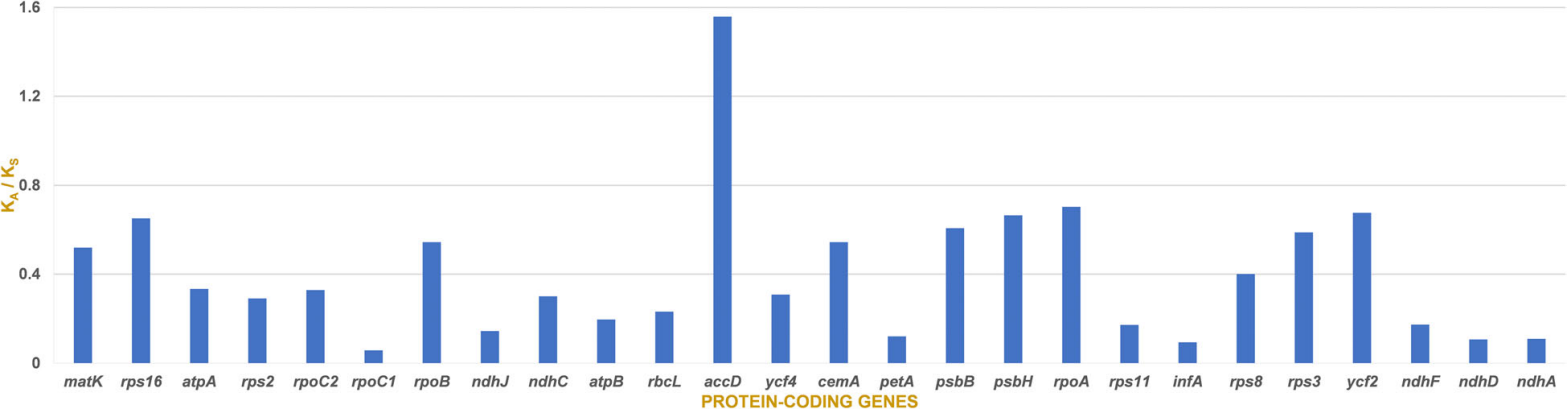
Comparison the ratio of non-synonymous (K_A_) to synonymous (K_S_) substitutions of 79 protein-coding genes of *Xanthium spinosum* vs. *X*. *sibiricum* cp genomes

The synonymous and nonsynonymous substitution rates were evaluated for 79 PCGs in the *X. spinosum* and *X*. *sibiricum* cp genomes. The K_A_/K_S_ ratios of nearly all genes were less than 1, except for the PCG *accD* (1.56) (Fig. 7).

### Positive selection analyses of the *accD* gene

Positive selection of the *accD* PCG in Heliantheae cp genome species was investigated using site-specific models with four comparisons (M0 vs. M3, M1 vs. M2a, M7 vs. M8, M8a vs. M8), using a likelihood ratio test (LRT) threshold of *p* ≤ 0.05 in EasyCodeML software. Among these models, M2a was the positive selective model and p (p_0_, p_1_ and p_2_) are the proportions of negative or purifying, neutral, and positive selection, respectively. The ω_2_ value of the *accD* gene was 3.70 in the M2a model. In addition, Bayes empirical Bayes (BEB) analyses were used to analyze the locations of consistent selective sites in the *accD* PCG using the M7 vs. M8 model comparison, and one site was found to potentially be under positive selection, with posterior probabilities greater than 0.95, while another site had probabilities greater than 0.99 (Table 3); the 2ΔLnL value was 25.91 and the *p-*value of LRT was 0 (Table 4).

**Table 3:** Comparison of site models, positive selective amino acid loci and estimation of parameters for *accD* protein-coding genes in the Heliantheae species

Protein-coding gene	Model	np	Ln L	Estimates of parameters				Model compared	LRT *P*-value	Positive sites
*accD*	M3	45	− 1567.812	p:	0.05791	0.85997	0.08211	M0 vs. M3	0	[]
				ω:	0.22724	0.22724	3.70209			
	M0	41	− 1597.602	ω0:	0.36846					Not Allowed
	M2a	44	−1567.812	p:	0.91789	0	0.08211	M1a vs. M2a	0.000196255	[]
				ω:	0.22724	1	3.70208			
	M1a	42	− 1576.349	p:	0.82957	0.17043				Not Allowed
				ω:	0.11911	1				
	M8	44	−1567.829	p0 = 0.91839	*p* = 29.41052	q = 99.00000		M7 vs. M8	0.000002362	35 S 0.936, 42 R 0.963*, 98 H 0.701, 129 Q 0.685, 177 S 0.616, 181 S 0.765, 184 N 0.519, 185 A 1.000**, 187 A 0.905
				(p1 = 0.08161)	ω = 3.72034					
	M7	42	− 1580.784	p=	0.07226	q=	0.08717			Not Allowed
	M8a	43	−1576.468	p0 = 0.82999	*p* = 13.67839	q = 99.00000		M8a vs. M8	0.000032257	Not Allowed
				(p1 = 0.17001)	ω = 1.00000					

Note:

[] – No data available

np represents the degree of freedom

Positively selected sites (* *p* > 95%; ** *p* > 99%)

**Table 4:** Comparison of likelihood ratio test (LRT) statistics of positive selection models against their null models (2ΔLnL) for the *accD* gene

Protein-coding genes	Comparison between models	2ΔLnL	*d.f.*	*p-*value
*accD*	M0 vs M3	59.579136	4	0
	M1 vs M2A	17.07219	2	0.0001963
	M7 vs M8	25.91159	2	0.0000024
	M8a vs M8	17.27995	1	0.0000323

### Phylogenetic analyses

**Fig. 8:**
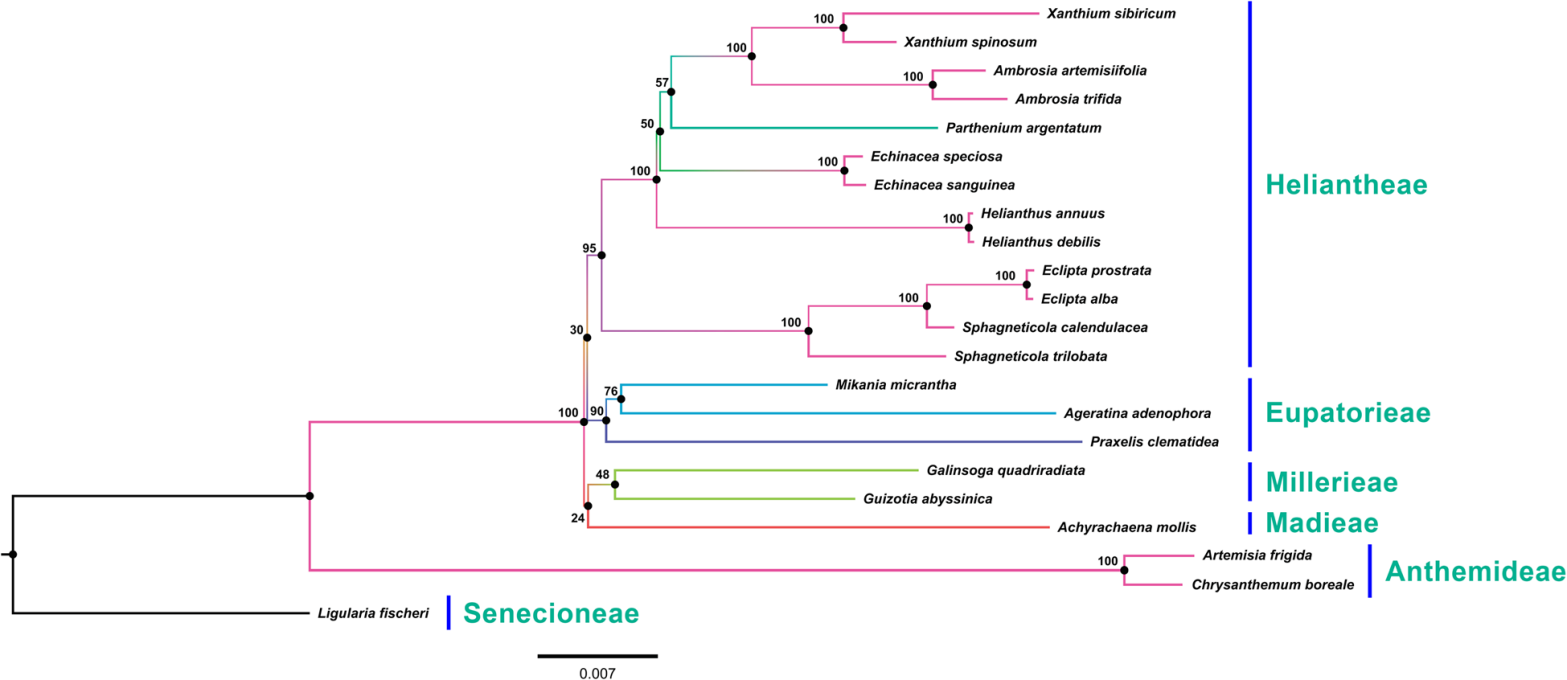
Molecular phylogenetic tree based on 79 protein-coding genes of 21 Asteraceae chloroplast genomes. *Achyrachaena mollis* set as the outgroup. The tree was constructed by maximum likelihood analysis of the conserved regions using the RAxML program and the GTRI nucleotide model. The stability of each tree node was tested by bootstrap analysis with 1000 replicates. Bootstrap values are indicated on the branches, and the branch length reflects the estimated number of substitutions per 1000 sites

In all, 71 PCGs from 21 cp genome sequences were selected for inferring phylogenetic relationships among closely related species of Heliantheae, and *Ligularia fischeri* (MG729822) was selected as an outgroup. A maximum likelihood tree was constructed using 71 concatenated PCGs in the cp genomes. The genus *Xanthium* was closely related to the genus *Ambrosia* (Fig. 8). Our analyses showed that *Parthenium* was a sister clade to both *Xanthium* and *Ambrosia*, and also an early-diverging lineage of the subtribe Ambrosiinae with a weak bootstrap value (57%).

## Discussion

The single circular cp genome structure of *X. spinosum* was similar to that of *X*. *sibiricum* with a typical quadripartite structure and equal GC content (37.45%) unevenly distributed across the cp genome. Relative to the LSC and SSC regions, the GC content is greater in IR regions across both cp genomes, possibly due to the presence of four extremely conserved rRNA genes with high GC content in these regions. The expansion and contraction of IR regions was the main cause of variation in cp genome size, and assessing these differences could shed light on the evolution of related taxa [30, 31]. The cp IR boundary regions of *X. spinosum* were compared to those of closely related species, and little difference was found, except for position changes in ѱ*ycf1*. The sizes of the four cp genomes (*X. spinosum*, *X*. *sibiricum*, *A. artemisiifolia*, and *P. argentatum*) were not affected. Moreover, the length of each region and the total genome size were similar to those of most plant cp genomes reported previously [32].

Repeat units, which are dispersed in cp genomes at high frequency, play a significant role in genome evolution [33–36]. Our comparative analyses of *X. spinosum* and *S. sibiricum* cp genomes showed a 24.4-fold higher level of repeats in *X. spinosum*. An earlier study reported that variation in the number and type of repeats may play a major role in plastome organization; however, we found no correlation between these large repeat regions and rearrangement endpoints [37]. SSRs, also known as microsatellite repeats [38, 39], are common in the cp genome, and these sequences display a high level of polymorphism, supporting their use as a genetic marker in previous investigations [40, 41]. The contents of different types of SSRs and their distributions among cp regions were similar in *X. spinosum* and *X*. *sibiricum*. Multiple definitions of repeat motifs and repeat number within motifs have been used in the literature; our SSR definition aligns with those of Bilgen et al. [42] and Karaca et al. [43].

The cp genomes of *Xanthium* showed less variation in non-coding regions than in their coding counterparts. The LSC region exhibited higher divergence levels than the IR and SSC regions (Fig. 6c). Specifically, the two IR regions were least divergent, perhaps due to the presence of four highly conserved rRNA sequences in those regions. The average nucleotide diversity (π) of intergenic regions was 0.0170, almost four times as high as that of PCGs (π = 0.004195), revealing that intergenic regions show greater divergence (Fig. 6d).

Not all PCGs are phylogenetically useful for determining taxonomic discrepancies [44]. In previous studies, several plastid and nuclear DNA markers from non-coding regions have been used to resolve the phylogenetic position of *Xanthium* species, leading to inconsistent results [11]. Hence, the use of the additional markers and broader taxonomic sampling are required to achieve greater phylogenetic resolution at low taxonomic levels [11, 45]. Therefore, in the present study, we proposed a set of 14 divergent regions between *X. spinosum* and *X*. *sibiricum* to resolve taxonomic discrepancies and provide a genetic barcode for the genus *Xanthium*. All of these regions are intergenic spacer regions, which might be useful for the development of molecular markers to use in phylogenetic and phylogeographic studies. The 14 sequences identified in the present study are extremely polymorphic compared to the sequences used in previous studies [6, 11, 45]. Based on our data, molecular markers can be developed for these intergenic regions that may be used for phylogenetic, phylogeographic, and barcoding studies of *Xanthium*. Moreover, this is the first report of the development of genetic markers based on these regions and their use to distinguish among *Xanthium* species. In addition, the nucleotide substitution rate and BEB analyses revealed that the *accD* gene may be under positive selection, and other positively selected sites detected in the present study may drive the *accD* PCG, supporting the occupation of various habitats [46, 47]. The earlier studies indicated that the gene *accD* encoded plastid beta carboxyl transferase subunit of acetyl-CoA carboxylase (ACCase) which is important for the proper chloroplast and as all stages of leaf growth [48], leaf longevity [49], fatty acid biosynthesis [50, 51] and embryo development [52]. Hence, the *accD* gene may have been involved in adaptation to specific ecological niches during the radiation of dicotyledonous plants [53].

Over the past few years, numerous plastid genome databases have been reported, offering an important foundation for resolving evolutionary, taxonomic, and phylogenetic questions in plants [54–60]. Our phylogenetic analyses showed that the genus *Xanthium* is most closely related to the genus *Ambrosia*. Several previous studies have used various methods including cladistic analyses [61, 62], cp restriction site variation assessments [63], and sequence analyses [11, 64] to understand the position of *Xanthium*, and these have shown that it is most closely related to *Ambrosia* species. Previous phylogenetic studies have shown that the genus *Parthenium* is an early-diverging lineage of the subtribe Ambrosiinae based on three plastid and two nuclear markers. We obtained consistent results, but with weak bootstrap support (57%). Somaratne et al. [6] suggested that *Parthenium* is not an early-diverging lineage of the subtribe Ambrosiinae, however, their phylogenetic analysis included only 46 cp PCGs. By contrast, we analyzed 71 PCGs in the present study, and the results suggest that *Parthenium* is an early-diverging lineage of subtribe Ambrosiinae.

## Conclusion

We aimed to expand the molecular genetic resources available for the species *X. spinosum* through high-throughput sequencing and cp genome assembly. The structural characteristics of the *X. spinosum* cp genome is similar to other angiosperms. However, fourteen highly variable regions were detected and suggested as potential markers for future barcoding and phylogenetic studies of *Xanthium* species. Hence, the sequence data for the complete *X. spinosum* cp genome could be used as to distinguish among *Xanthium* species and resolve the phylogenetic relationships within the Ambrosiinae lineage.

## Methods

### DNA extraction and sequencing of *Xanthium spinosum*

Leaf material of *Xanthium spinosum* was obtained from Dr. George A Yatskievych, Curator, Plant Resources Center, University of Texas Herbarium (19–056), Austin, Texas, USA. Total genomic DNA was extracted using a modified cetyltrimethylammonium bromide method [65]. Illumina sequencing was carried out by LabGenomics, Seongnam, South Korea, using the Illumina HiSeq 2500 sequencing system. A paired-end library (150 × 2) was constructed with an insert size of 350 base pairs (bp). Read quality was analyzed with FastQC v0.11.9 [66] and low-quality reads were removed with Trimmomatic 0.39 [67]. The resultant clean reads were filtered using the GetOrganelle v1.6.0 pipeline (https://github.com/Kinggerm/GetOrganelle) to obtain plastid-like reads, and then the filtered reads were assembled de novo using SPAdes v3.12.0 [68]. The complete cp genome sequence of *X. spinosum* and its gene annotation were submitted to GenBank (MT668935).

### Annotation of *X. spinosum* cp genome

The online program Dual Organellar GenoMe Annotator (DOGMA) was used to annotate the cp genome sequence of *X. spinosum* [69]. The initial annotation, putative starts, stops, and intron positions were fine-tuned through comparison with homologous genes in the closely related species *X*. *sibiricum* [6]. Transfer RNA genes were validated using tRNAscan-SE v1.21 with the default settings [70]. The program OGDRAW v1.3.1 was employed to draw a circular map of the *X. spinosum* cp genome [71].

### Comparative cp genome analyses

The mVISTA program, which uses the Shuffle-LAGAN model, was employed to compare the cp genome of *X. spinosum* with three closely related cp genomes from *X*. *sibiricum*, *Ambrosia artemisiifolia*, and *Parthenium argentatum* using the *X. spinosum* annotation as a reference [72]. The boundaries between IR and SC regions of these species were also compared and investigated.

### Repeat sequence and simple sequence repeats (SSRs) analyses

The program REPuter was used to predict the presence of repeat sequences in the *X. spinosum* and *X*. *sibiricum* cp genomes, including forward, reverse, palindromic, and complementary repeats [73]. The following parameters were used to identify repeats with REPuter: Hamming distance 3, minimum sequence identity of 90%, and repeat size > 30 bp. Phobos software v1.0.6 was employed to identify SSRs in the *X. spinosum* and *X*. *sibiricum* cp genomes; the match, mismatch, gap, and N positions parameters were set to 1, − 5, − 5, and 0, respectively [74]. For repeat and SSR marker analyses, only one IR region was used.

### Anaglyses of genetic divergence

To analyze genetic divergence, the PCGs, intergenic, and intron-containing regions of the *X. spinosum* and *X*. *sibiricum* cp genomes were extracted and aligned independently using Geneious Prime v2020.1.2 (Biomatters, New Zealand). Genetic divergence between these *Xanthium* species was calculated based on nucleotide diversity (π) and the total number of polymorphic sites using DnaSP v5.10.01 [75]. For this analysis, gaps and missing data were excluded.

### Characterization of substitution rates

To calculate the synonymous (K_S_) and nonsynonymous (K_A_) substitution rates, the cp genome of *X. spinosum* was compared to that of *X*. *sibiricum*. Corresponding single-functional PCG exons were extracted from both genomes and aligned independently using Geneious Prime v2020.1.2 (Biomatters, New Zealand). The aligned sequences were translated into protein sequences and analyzed using DnaSP v5.10.01 to obtain K_A_ and K_S_ substitution rates without stop codons.

### Positive selection analyses

Positive selection (M2a and M8) and control (M1a, M7, and M8a) models provided in EasyCodeML software v1.21 [76] were used to identify the occurrence of positive selection (ω > 1) on the *accD* locus in Heliantheae cp genomes. The sequence of the *accD* gene was aligned using the program MAFFT v1.4.0 [77], and the maximum likelihood phylogenetic tree was constructed using RAxML v7.2.6 [78]. The site-specific model was used to calculate nonsynonymous (K_A_) and synonymous substitution (K_S_) rates using EasyCodeML. The codon substitution models M0, M1a, M2a, M3, M7, M8, and M8a were analyzed. The likelihood ratio test was used to identify positively selected sites in comparisons of M0 (one-ratio) vs. M3 (discrete), M1a (neutral) vs. M2a (positive selection), M7 (β) vs. M8 (β and ω > 1) and M8a ((β and ω = 1) vs. M8 using a site-specific model [76]. The likelihood ratio test (LRT) for these comparisons was used to evaluate the selection strength and *p*-values of less than 0.05 from the chi-square (χ^2^) test were considered significant. If the LRT *p*-values were significant (< 0.05), the Bayes Empirical Bayes (BEB) method was implemented to identify codons under positive selection. BEB values higher than 0.95 and 0.99 indicate sites that are potentially under positive selection and highly positive selection, respectively.

### Phylogenetic tree analyses

A phylogenetic tree was constructed using 71 PCGs from 21 Asteroideae cp genomes, with *L. fischeri* as the outgroup. A total of 20 complete cp genome sequences were downloaded from the NCBI Organelle Genome Resource database. The aligned PCG sequences were saved in PHYLIP format using Clustal X v2.1 [79], and phylogenetic analyses were conducted based on the maximum likelihood (ML) method and the GTRI model using RAxML v7.2.6 with 1000 bootstrap replications [78].

## Data Availability

The dataset generated and or analysed during the current study is deposited in the genebank with accession number: MT668935. The phylogenetic genome datasets used and analysed in this study were retrieved from the National Center for Biotechnology Information Organelle Genome Resource Database.

## Abbreviations

cp: Chloroplast
LSC: Large single-copy
SSC: Small single-copy
IR: Inverted-repeat
tRNA: Transfer RNA
rRNA: Ribosomal RNA
K_S_: Synonymous substitution
K_A_: Nonsynonymous substitution
ω: Nonsynonymous vs. synonymous ratio
SSR: Simple sequence repeats
LRT: Likelihood ratio test
π: Nucleotide diversity

## References

[1] Daniell H, Lin CS, Yu M, Chang WJ. Chloroplast genomes: diversity, evolution, and applications in genetic engineering. Genome Biol. 2016;17:134. 10.1186/s13059-016-1004-2 PMC4918201 27339192

[2] Palmer JD (1985). Comparative organization of chloroplast genomes. Annu Rev Genet.

[3] Moore MJ, Dhingra A, Soltis PS, Shaw R, Farmerie WG, Folta KM, Soltis DE. Rapid and accurate pyrosequencing of angiosperm plastid genomes. BMC Plant Biol. 2006;6:17. 10.1186/1471-2229-6-17 PMC1564139 16934154

[4] Williams AV, Miller JT, Small I, Nevill PG, Boykin LM (2016). Integration of complete chloroplast genome sequences with small amplicon datasets improves phylogenetic resolution in *Acacia*. Mol Phylogenet Evol.

[5] Liu Q, Li X, Li M, Xu W, Schwarzacher T, Heslop-Harrison JS (2020). Comparative chloroplast genome analyses of *Avena*: insights into evolutionary dynamics and phylogeny. BMC Plant Biol.

[6] Somaratne Y, Guan D-L, Wang W-Q, Zhao L, Xu S-Q (2019). Complete chloroplast genome sequence of *Xanthium sibiricum* provides useful DNA barcodes for future species identification and phylogeny. Plant Syst Evol.

[7] Amin S, Khan H (2016). Revival of natural products: utilization of modern technologies. Curr Bioactive Compounds.

[8] Robbins W (1940). Alien plants growing without cultivation in California. Bulletin Calif Agric Exp Station.

[9] Holm L, Pluncknett D, Pancho J, Herberger J (1977). The world's worst weeds.

[10] Munz P, Keck D (1973). A California flora and supplement.

[11] Tomasello S, Heubl G (2017). Phylogenetic analysis and molecular characterization of *Xanthium sibiricum* using DNA barcoding, PCR-RFLP, and specific primers. Planta Med.

[12] Romero M, Zanuy M, Rosell E, Cascante M, Piulats J, Font-Bardia M, Balzarini J, De Clerq E, Pujol MD (2015). Optimization of xanthatin extraction from *Xanthium spinosum* L. and its cytotoxic, anti-angiogenesis and antiviral properties. Eur J Med Chem.

[13] Salinas A (1998). Ruiz RELd, Ruiz SO: sterols, flavonoids, and sesquiterpenic lactones from *Xanthium spinosum* (Asteraceae). Acta Farm Bonaer.

[14] Ginesta-Peris E, Garcia-Breijo FJ, Primo-Yufera E (1993). Antimicrobial activity of xanthatin from *Xanthium spinosum* L. Lett Appl Microbiol.

[15] Yoon JH, Lim HJ, Lee HJ, Kim HD, Jeon R, Ryu JH (2008). Inhibition of lipopolysaccharide-induced inducible nitric oxide synthase and cyclooxygenase-2 expression by xanthanolides isolated from *Xanthium strumarium*. Bioorg Med Chem Lett.

[16] Willians RH, Martin FB, Henley ED, Swanson HE (1959). Inhibitors of insulin degradation, metabolism. Clin Exp Rheumatol.

[17] Naidenova E, Kolarova-Pallova I, Popov D, Dimitrova-Konaklieva S, Dryanovska-Noninska L (1988). Isolation and obtaining of sesquiterpene lactones with antitumor properties – xanthinin, stizolicin, and solstitialin. Natl Oncol Cent Med Acad.

[18] Cunat P, Primo E, Sanz I, Garcera MD, March MC, Bowers WS, Martinez-Pardo R (1990). Biocidal activity of some Spanish Mediterranean plants. J Agric Food Chem.

[19] Turnne CY, Sanche SE, Hoban DJ, Karylowsky JA, Kabani AM (1999). Rapid identification of fungi by using the ITS2 genetic region and an automated fluorescent capillary electrophoresis system. J Clin Microbiol.

[20] Trobajo R, Mann DG, Clavero E, Evans KM, Vanormelingen P, McGregor RC (2010). The use of partial *cox1*, *rbcL* and LSU rDNA sequences for phylogenetics and species identification within the *Nitzschia palea* species complex (Bacillariophyceae). Eur J Phycol.

[21] Liu C, Liang D, Gao T, Pang X, Song J, Yao H, Han J, Liu Z, Guan X, Jiang K (2011). PTIGS-IdIt, a system for species identification by DNA sequences of the *psbA*-*trnH* intergenic spacer region. BMC Bioinformatics.

[22] Millspaugh C, Sherff E (1919). Revision of the north American species of *Xanthium*. Field Mus Nat Hist Zool Ser.

[23] Widder F: Die Arten der Gattung *Xanthium*. Beiträge zu einer Monographie. Repert Spec Nov Regn Veget 1923, 20:1–223.

[24] Löve D, Dansereau L (1959). Biosystematic studies on *Xanthium*: taxonomic appraisal and ecological status. Can J Bot.

[25] Strother J (2006). *Xanthium*. Flora of North America.

[26] Hollingsworth PM, Graham SW, Little DP. Choosing and using a plant DNA barcode. PLoS One. 2011;6(5):e19254. 10.1371/journal.pone.0019254 PMC3102656 21637336

[27] Sanita Lima M, Woods LC, Cartwright MW, Smith DR (2016). The (in) complete organelle genome: exploring the use and nonuse of available technologies for characterizing mitochondrial and plastid chromosomes. Mol Ecol Resour.

[28] Kumar S, Hahn FM, McMahan CM, Cornish K, Whalen MC. Comparative analysis of the complete sequence of the plastid genome of *Parthenium argentatum* and identification of DNA barcodes to differentiate *Parthenium* species and lines. BMC Plant Biol. 2009;9. 10.1186/1471-2229-9-131 PMC2784773 19917140

[29] Amiryousefi A, Hyvonen J, Poczai P (2017). The plastid genome sequence of the invasive plant common ragweed (*Ambrosia artemisiifolia*, Asteraceae). Mitochondrial DNA B.

[30] Boudreau E, Turmel M (1995). Gene rearrangements in *Chlamydomonas* chloroplast DNAs are accounted for by inversions and by the expansion/contraction of the inverted repeat. Plant Mol Biol.

[31] Nazareno AG, Carlsen M, Lohmann LG. Complete chloroplast genome of *Tanaecium tetragonolobum*: the first bignoniaceae plastome. PLoS One. 2015;10(6):e0129930. 10.1371/journal.pone.0129930 PMC4478014 26103589

[32] Zhang X, Zhou T, Kanwal N, Zhao YM, Bai GQ, Zhao GF. Completion of eight *Gynostemma* BL. (Cucurbitaceae) chloroplast genomes: Characterization, comparative analysis, and phylogenetic relationships. Front Plant Sci. 2017;8:1583. 10.3389/fpls.2017.01583 PMC5600969 28955369

[33] Dong WP, Xu C, Cheng T, Lin K, Zhou SL (2013). Sequencing angiosperm plastid genomes made easy: a complete set of universal primers and a case study on the phylogeny of Saxifragales. Genome Biol Evol.

[34] Liu WZ, Kong HH, Zhou J, Fritsch PW, Hao G, Gong W. Complete chloroplast genome of *Cercis chuniana* (Fabaceae) with structural and genetic comparison to six species in Caesalpinioideae. Int J Mol Sci. 2018;19(5):1286. 10.3390/ijms19051286 PMC5983592 29693617

[35] Xie DF, Yu Y, Deng YQ, Li J, Liu HY, Zhou SD, He XJ. Comparative analysis of the chloroplast genomes of the Chinese endemic genus *Urophysa* and their contribution to chloroplast phylogeny and adaptive evolution. Int J Mol Sci. 2018;19(7):1847. 10.3390/ijms19071847 PMC6073864 29932433

[36] Shi HW, Yang M, Mo CM, Xie WJ, Liu C, Wu B, Ma XJ. Complete chloroplast genomes of two *Siraitia* merrill species: Comparative analysis, positive selection and novel molecular marker development. PLoS One. 2019;14(12):e0226865. 10.1371/journal.pone.0226865 PMC6924677 31860647

[37] Liu Y, Huo NX, Dong LL, Wang Y, Zhang SX, Young HA, Feng XX, Gu YQ. Complete chloroplast genome sequences of Mongolia medicine *Artemisia frigida* and phylogenetic relationships with other plants. PLoS One. 2013;8(2):e57533. 10.1371/journal.pone.0057533 PMC3583863 23460871

[38] Ince AG, Karaca M, Elmasulu SY (2014). New microsatellite and CAPS-microsatellite markers for clarifying taxonomic and phylogenetic relationships within *Origanum* L. Mol Breed.

[39] Ince AG, Karaca M, Onus AN (2009). Polymorphic microsatellite markers transferable across *Capsicum* species. Plant Mol Biol Report.

[40] Smith JSC, Chin ECL, Shu H, Smith OS, Wall SJ, Senior ML, Mitchell SE, Kresovich S, Ziegle J (1997). An evaluation of the utility of SSR loci as molecular markers in maize (*Zea mays* L.): comparisons with data from RFLPS and pedigree. Theoritical applied Genetics.

[41] Kawabe A, Nukii H, Furihata HY. Exploring the history of chloroplast capture in *Arabis* using whole chloroplast genome sequencing. Int J Mol Sci. 2018;19(2):602. 10.3390/ijms19020602 PMC5855824 29463014

[42] Karaca M, Bilgen M, Onus AN, Ince AG, Elmasulu SY (2005). Exact tandem repeats analyzer (E-TRA): a new program for DNA sequence mining. J Genet.

[43] Bilgen M, Karaca M, Onus AN, Ince AG (2004). A software program combining sequence motif searches with keywords for finding repeats containing DNA sequences. Bioinformatics.

[44] Pfeil BE, Brubaker CL, Craven LA, Crisp MD (2002). Phylogeny of *Hibiscus* and the tribe Hibisceae (Malvaceae) using chloroplast DNA sequences of *ndhF* and the *rpl16* intron. Syst Bot.

[45] Wallace LJ, Boilard SMAL, Eagle SHC, Spall JL, Shokralla S, Hajibabaei M (2012). DNA barcodes for everyday life: routine authentication of natural health products. Food Res Int.

[46] Hu QM, Zhu Y, Liu Y, Wang N, Chen SL. Cloning and characterization of *wnt4a* gene and evidence for positive selection in half-smooth tongue sole (*Cynoglossus semilaevis*). Sci Rep-Uk. 2014;4:7167. 10.1038/srep07167 PMC4241513 25418599

[47] Dhar D, Dey D, Basu S, Fortunato H. Understanding the adaptive evolution of mitochondrial genomes in intertidal chitons. bioRxiv. 2020; 10.1101/2020.03.06.980664.

[48] Kode V, Mudd EA, Iamtham S, Day A (2005). The tobacco plastid *accD* gene is essential and is required for leaf development. Plant J.

[49] Madoka Y, Tomizawa K, Mizoi J, Nishida I, Nagona Y, Sasaki Y (2002). Chloroplast transformation with modified *accD* operon increases acetyl-CoA carboxylase and causes extension of leaf longevity and increase in seed yield in tobacco. Plant Cell Physiol.

[50] Ohlrogge J, Browse J (1995). Lipid biosynthesis. Plant Cell.

[51] Sasaki Y, Nagano Y (2004). Plant acetyl-CoA carboxylase: structure, biosynthesis, regulation, and gene manipulation for plant breeding. Biosci Biotechnol Biochem.

[52] Bryant N, Lloyd J, Sweeney C, Myouga F, Meinke D (2011). Identification of nuclear genes encoding chloroplast-localized proteins required for embryo development in *Arabidopsis*. Plant Physiol.

[53] Hu S, Sablok G, Wang B, Qu D, Barbaro E, Viola R (2015). Plastome organization and evolution of chloroplast genes in *Cardamine* species adapted to contrasting habitats. BMC Genomics.

[54] Boudreau E, Takahashi Y, Lemieux C, Turmel M, Rochaix JD (1997). The chloroplast *ycf3* and *ycf4* open reading frames of *Chlamydomonas reinhardtii* are required for the accumulation of the photosystem I complex. EMBO J.

[55] Yang JB, Tang M, Li HT, Zhang ZR, Li DZ. Complete chloroplast genome of the genus *Cymbidium*: lights into the species identification, phylogenetic implications and population genetic analyses. BMC Evol Biol. 2013;13. 10.1186/1471-2148-13-84 PMC3644226 23597078

[56] Raman G, Park S. The complete chloroplast genome sequence of *Ampelopsis*: gene organization, comparative analysis, and phylogenetic relationships to other angiosperms. Front Plant Sci. 2016;7. 10.3389/fpls.2016.00341 PMC4800181 27047519

[57] Zhang YJ, Du LW, Liu A, Chen JJ, Wu L, Hu WM, Zhang W, Kim K, Lee SC, Yang TJ, et al. The complete chloroplast genome sequences of five *Epimedium* species: lights into phylogenetic and taxonomic analyses. Front Plant Sci. 2016;7. 10.3389/fpls.2016.00306 PMC4791396 27014326

[58] Kahraman K, Lucas SJ. Comparison of different annotation tools for characterization of the complete chloroplast genome of *Corylus avellana* cv Tombul. BMC Genomics. 2019;20(1):874. 10.1186/s12864-019-6253-5 PMC6865063 31747873

[59] Li XQ, Zuo YJ, Zhu XX, Liao S, Ma JS. Complete chloroplast genomes and comparative analysis of sequences evolution among seven *Aristolochia* (Aristolochiaceae) medicinal species. Int J Mol Sci. 2019;20(5):1045. 10.3390/ijms20051045 PMC6429227 30823362

[60] Li CJ, Wang RN, Li DZ. Comparative analysis of plastid genomes within the Campanulaceae and phylogenetic implications. PLoS One. 2020;15(5):e0233167. 10.1371/journal.pone.0233167 PMC7224561 32407424

[61] Karis P (1993). Heliantheae sensu lato (Asteraceae), clades and classification. Plant Syst Evol.

[62] Karis P (1995). Cladistics of the subtribe Ambrosiinae (Asteraceae: Heliantheae). Syst Bot.

[63] Miao B, Turner B, Mabry T (1995). Systematic implications of chloroplast DNA variation in the subtribe Ambrosiinae (Asteraceae: Heliantheae). Am J Bot.

[64] Martin MD, Quiroz-Claros E, Brush GS, Zimmer EA (2018). Herbarium collection-based phylogenetics of the ragweeds (Ambrosia, Asteraceae). Mol Phylogenet Evol.

[65] Doyle J (1990). Isolation of plant DNA from fresh tissue. Focus.

[66] Andrews S: FASTQC. A quality control tool for high throughput sequence data 2010.

[67] Bolger AM, Lohse M, Usadel B (2014). Trimmomatic: a flexible trimmer for Illumina sequence data. Bioinformatics.

[68] Nurk S, Bankevich A, Antipov D, Gurevich AA, Korobeynikov A, Lapidus A, Prjibelski AD, Pyshkin A, Sirotkin A, Sirotkin Y (2013). Assembling single-cell genomes and mini-metagenomes from chimeric MDA products. J Comput Biol.

[69] Wyman SK, Jansen RK, Boore JL (2004). Automatic annotation of organellar genomes with DOGMA. Bioinformatics.

[70] Schattner P, Brooks AN, Lowe TM (2005). The tRNAscan-SE, snoscan and snoGPS web servers for the detection of tRNAs and snoRNAs. Nucleic Acids Res.

[71] Lohse M, Drechsel O, Bock R (2007). OrganellarGenomeDRAW (OGDRAW): a tool for the easy generation of high-quality custom graphical maps of plastid and mitochondrial genomes. Curr Genet.

[72] Frazer KA, Pachter L, Poliakov A, Rubin EM, Dubchak I (2004). VISTA: computational tools for comparative genomics. Nucleic Acids Res.

[73] Kurtz S, Choudhuri JV, Ohlebusch E, Schleiermacher C, Stoye J, Giegerich R (2001). REPuter: the manifold applications of repeat analysis on a genomic scale. Nucleic Acids Res.

[74] Mayer C, Leese F, Tollrian R (2010). Genome-wide analysis of tandem repeats in *Daphnia pulex*--a comparative approach. BMC Genomics.

[75] Librado P, Rozas J (2009). DnaSP v5: a software for comprehensive analysis of DNA polymorphism data. Bioinformatics.

[76] Gao F, Chen C, Arab DA, Du Z, He Y, Ho SYW (2019). EasyCodeML: a visual tool for analysis of selection using CodeML. Ecol Evol.

[77] Katoh K, Standley DM (2013). MAFFT multiple sequence alignment software version 7: improvements in performance and usability. Mol Biol Evol.

[78] Stamatakis A, Hoover P, Rougemont J (2008). A rapid bootstrap algorithm for the RAxML web servers. Syst Biol.

[79] Larkin MA, Blackshields G, Brown NP, Chenna R, McGettigan PA, McWilliam H, Valentin F, Wallace IM, Wilm A, Lopez R (2007). Clustal W and Clustal X version 2.0. Bioinformatics.

