# Correction to: Characteristics of the completed chloroplast genome sequence of *Xanthium spinosum*: comparative analyses, identification of mutational hotspots and phylogenetic implications

**DOI:** 10.1186/s12864-021-07392-w

**Published:** 2021-02-01

**Authors:** Gurusamy Raman, Kyu Tae Park, Joo-Hwan Kim, SeonJoo Park

**Affiliations:** 1 Department of Life Sciences, Yeungnam University, Gyeongsan, Gyeongsangbuk-do Republic of Korea 38541; 2 Department of Life Science, Gachon University, Seongnam, Gyeonggi-do, Republic of Korea

**Correction to: BMC Genomics 21, 855 (2020)**

**https://doi.org/10.1186/s12864-020-07219-0**

Following publication of the original article [1], the authors identified an error in an author’s name.

The incorrect name was: JooHwan Kim

The correct author name is: Joo-Hwan Kim

The author group has been updated above and the original article [1] has been corrected.
